# 2256. Racial and Socioeconomic Disparities Evident in Inappropriate Antibiotic Prescribing in the Emergency Department

**DOI:** 10.1093/ofid/ofad500.1878

**Published:** 2023-11-27

**Authors:** Eili Klein, Mustapha O Saheed, Nathan A Irvin, Kamna S Balhara, Oluwakemi Badaki-Makun, Suprena E Z Poleon, Gabor Kelen, Sara E Cosgrove, Jeremiah S Hinson

**Affiliations:** Johns Hopkins School of Medicine, Baltimore, Maryland; Johns Hopkins, Baltimore, MD; Johns Hopkins University School of Medicine, Elkridge, Maryland; Johns Hopkins University School of Medicine, Elkridge, Maryland; Johns Hopkins School of Medicine, Baltimore, Maryland; Center for Disease Dynamics Economics and Policy, Fort Worth, Texas; Johns Hopkins, Baltimore, MD; Johns Hopkins School of Medicine, Baltimore, Maryland; Johns Hopkins University School of Medicine, Elkridge, Maryland

## Abstract

**Background:**

Inappropriate antibiotic prescribing for acute respiratory infections is a common source of low-value care in the emergency department. Racial disparities have been noted in episodes of low-value care, particularly in children, but the intersection between race and socioeconomics in inappropriate prescribing has received less attention. Decisions to prescribe antibiotics outside of guideline recommendations may result from underlying bias. We evaluated inappropriate prescribing by race and socioeconomic status in five emergency departments of a large hospital system.

**Methods:**

We conducted an observational analysis of antibiotic prescribing in adult and pediatric EDs at five hospitals between October 2015 and March 2023. We calculated the rate of inappropriate prescribing for ED encounters that had an ICD-10 for an acute respiratory tract infection (ARTI) that did not require antibiotics and that had no secondary ICD-10 code on the record for which antibiotics may be indicated. Multivariable regression analysis controlling for comorbidities and patient-level factors was used to estimate the odds ratio of prescribing.

**Results:**

A total of 147,402 ED encounters were included, of which ∼50% identified as Black, 23% as White, and 15% as Hispanic. Inappropriate prescribing rates were 8.5% overall, 13.6% for White patients, 7% for Black patients and 6% for Hispanic patients. After adjusting for patient-level factors, including socioeconomic status,White patients had 1.39 (95% CI, 1.32-1.47) higher odds of receiving an inappropriate prescription compared to Black patients. Patients residing in areas of greater socioeconomic deprivation, regardless of race, had 0.70 (95% CI, 0.66-0.75) lower odds of receiving a prescription.

Inappropriate Prescribing Rate by Year and Race

**Figure:**
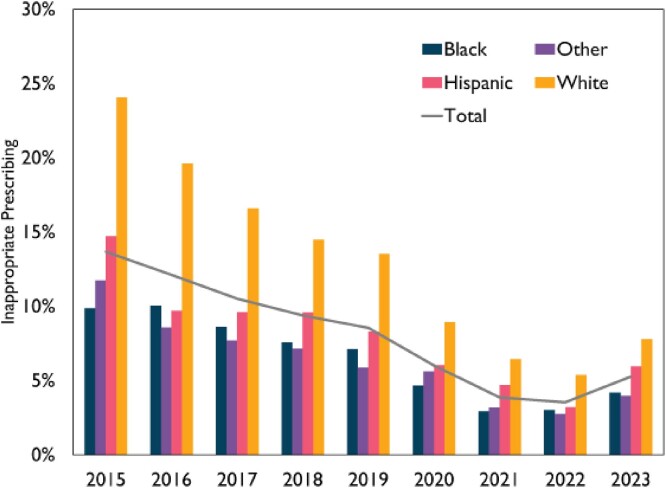

The overall rate of inappropriate prescribing did fall over the years, both overall (black line) and by race, but differences in prescribing by race persisted.

Odds ratios of a patient being prescribed an inappropriate antibiotic

**Figure:**
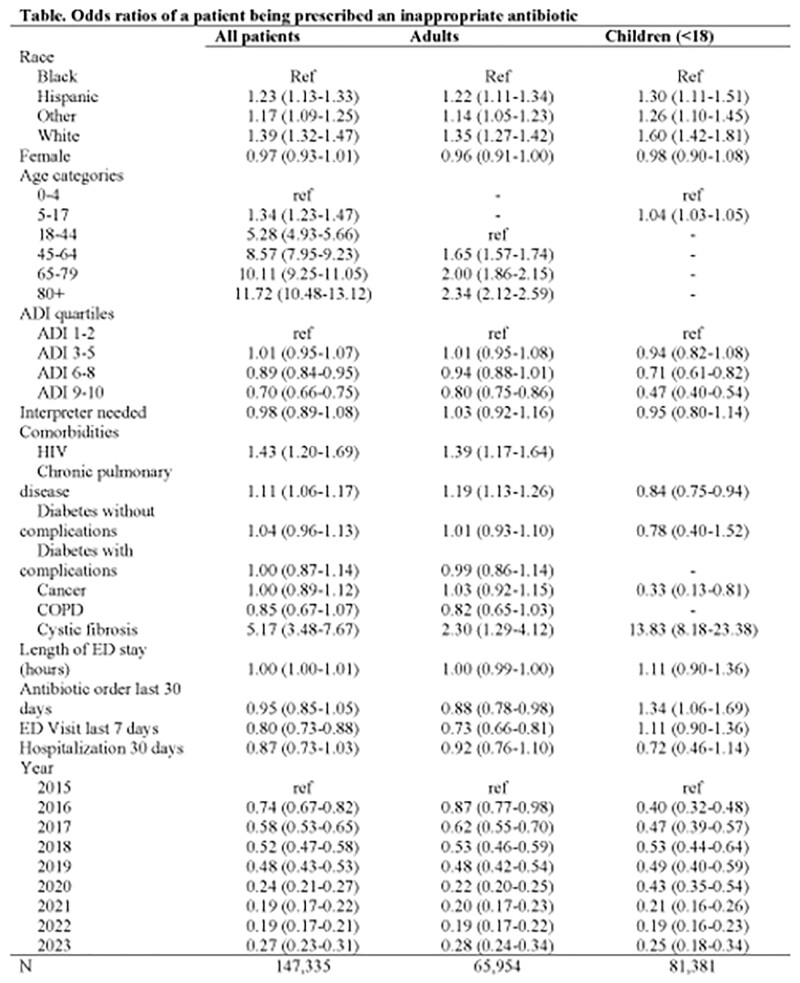

**Conclusion:**

Our results suggest that after controlling for patient-level factors, White patients and patients from wealthier areas are more likely to receive inappropriate antibiotic treatment. While likely multifactorial in origin, it is possible that these disparities are driven by clinicians’ implicit biases that lead to overtreatment of White and wealthier patients. Further qualitative and quantitive approaches are needed to elucidate mechanisms and solutions to these disparities in care.

**Disclosures:**

**Oluwakemi Badaki-Makun, MD, PhD**, Beckman Coulter: Grant/Research Support **Sara E. Cosgrove, MD, MS**, Debiopharm: Advisor/Consultant|Duke Clinical Research Institute: Advisor/Consultant **Jeremiah S. Hinson, MD, PhD**, Beckman Coulter: Advisor/Consultant|Beckman Coulter: Grant/Research Support|Beckman Coulter: TriageGO

